# Probing the Matter: A Case of Rectal Foreign Body Insertion in a Patient With Intellectual Disability

**DOI:** 10.7759/cureus.77139

**Published:** 2025-01-08

**Authors:** Shalmali Dharmadhikari, Akshay Rathod, Girish Bakhshi

**Affiliations:** 1 Department of General Surgery, Grant Medical College and Sir JJ group of Hospitals, Mumbai, IND; 2 Department of General Surgery, Grant Medical College and Sir JJ Group of Hospitals, Mumbai, IND

**Keywords:** dangerous sexual practices, intellectual disability, mental health awareness, rectal foreign body insertion, sexual gratification

## Abstract

Rectal foreign body insertion presents a challenging clinical scenario, with increasing incidence, particularly among males. In both acute management and long-term care, particularly when associated with psychiatric illness or intellectual disability, careful consideration is required. We report a 23-year-old male with mild intellectual disability who presented with a retained handheld bidet shower inserted for sexual gratification. He had a similar episode within the past six months. Despite the object being partially visible externally - that may cause the inexperienced, unassuming physician to falsely believe the ease of removal - it is imperative to follow proper diagnostic and management protocols for safe retrieval. Initial evaluation included radiographic assessment by X-ray imaging to determine the object's position and rule out complications. Successful transanal extraction was achieved without complications under general anesthesia. Postoperative CT with rectal contrast confirmed the absence of perforation or lingering damage related to the foreign body or procedure. The patient was subsequently referred for psychiatric evaluation and started on olanzapine but was later lost to follow-up.

This case highlights the importance of systematic management protocols in rectal foreign body extraction, regardless of the apparent simplicity of retrieval. It also emphasizes the need for comprehensive psychiatric evaluation and follow-up care, particularly in patients with intellectual disability, while addressing the persistent social stigma surrounding both rectal foreign body insertion and mental health issues.

## Introduction

Rectal foreign body insertions represent a growing healthcare challenge, with recent epidemiological data showing an increasing temporal trend and marked male predominance [1]. According to a study in the United States, the annual incidence has risen from 1.2 to 1.9 per 100,000 persons between 2012 and 2021, with cases dating back to the 16th century [2,3]. While anal eroticism remains the most common motivation [1], these presentations become particularly complex in the context of psychiatric illness or intellectual disability. A retrospective analysis reported that 37.5% of patients presenting with rectal foreign bodies had concurrent psychiatric illness [4]. These cases often demonstrate repetitive patterns of behavior and may be linked to various psychiatric conditions including schizophrenia, depression, and psychosis. Management of such cases requires a delicate balance between urgent medical intervention and sensitive patient care. The physician-patient relationship is particularly crucial, requiring an unprejudiced stance while maintaining patient dignity and trust. This case report presents an instance of rectal foreign body insertion in a young adult with intellectual disability, highlighting several important aspects: the relationship between psychiatric conditions and foreign body insertion (including swallowed and inhaled), the importance of following proper management protocols even when extraction appears straightforward, the taboo nature of these acts, the lack of mental health awareness, the reluctance to seek treatment for both these situations, and current management guidelines.

## Case presentation

**Figure 1:**
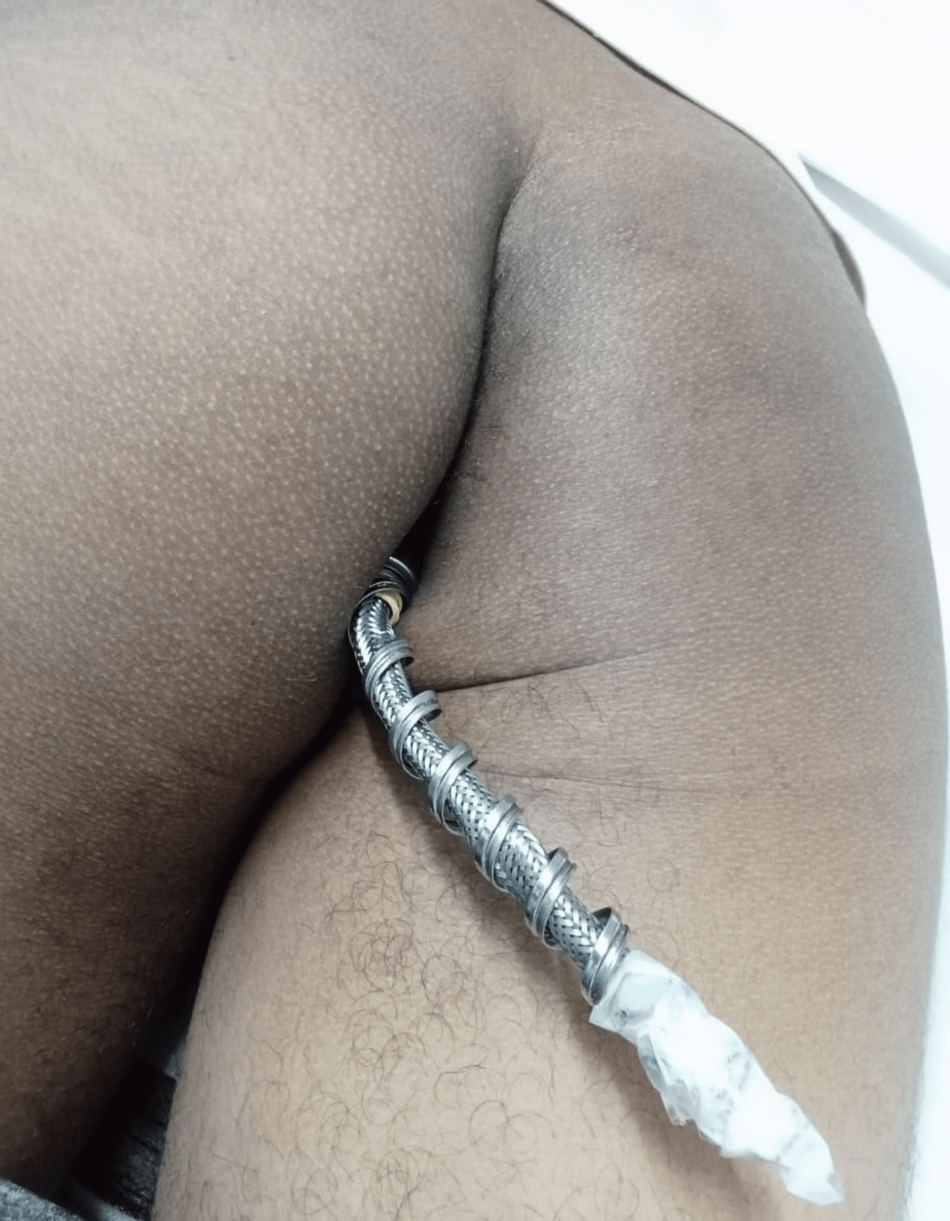
Shower bidet pipe protruding from the anal orifice.

We present a case of a 23-year-old male with mild intellectual disability, as reported by his parents, who observed gradually decreasing learning abilities and communication skills throughout his preschool and school years but did not seek treatment or assistance (not documented and not on any medications). He presented to the emergency room in the early hours of the morning with complaints of pain and discomfort due to the voluntary insertion of a handheld bidet shower into the anal orifice the previous night. This was the second episode within six months. The first time, the patient managed to remove it himself. However, this time, he was unsuccessful in his efforts. The bidet shower had traversed the whole lumen, in its entirety, with part of the shower bidet pipe remaining outside the anus. After waiting the whole night and trying to remove it in vain, a plumber was called to cut the attached pipe the following morning. The patient presented with pain and distress and provided a self-reported history but avoided eye contact. He was alert to time, place, and person, cognitively intact, and confessed to performing these acts for sexual pleasure. He was vitally stable with an unremarkable systemic examination. On local examination, the transected pipe of the shower bidet that remained outside the anus was evident (Figure 1). 

**Figure 2:**
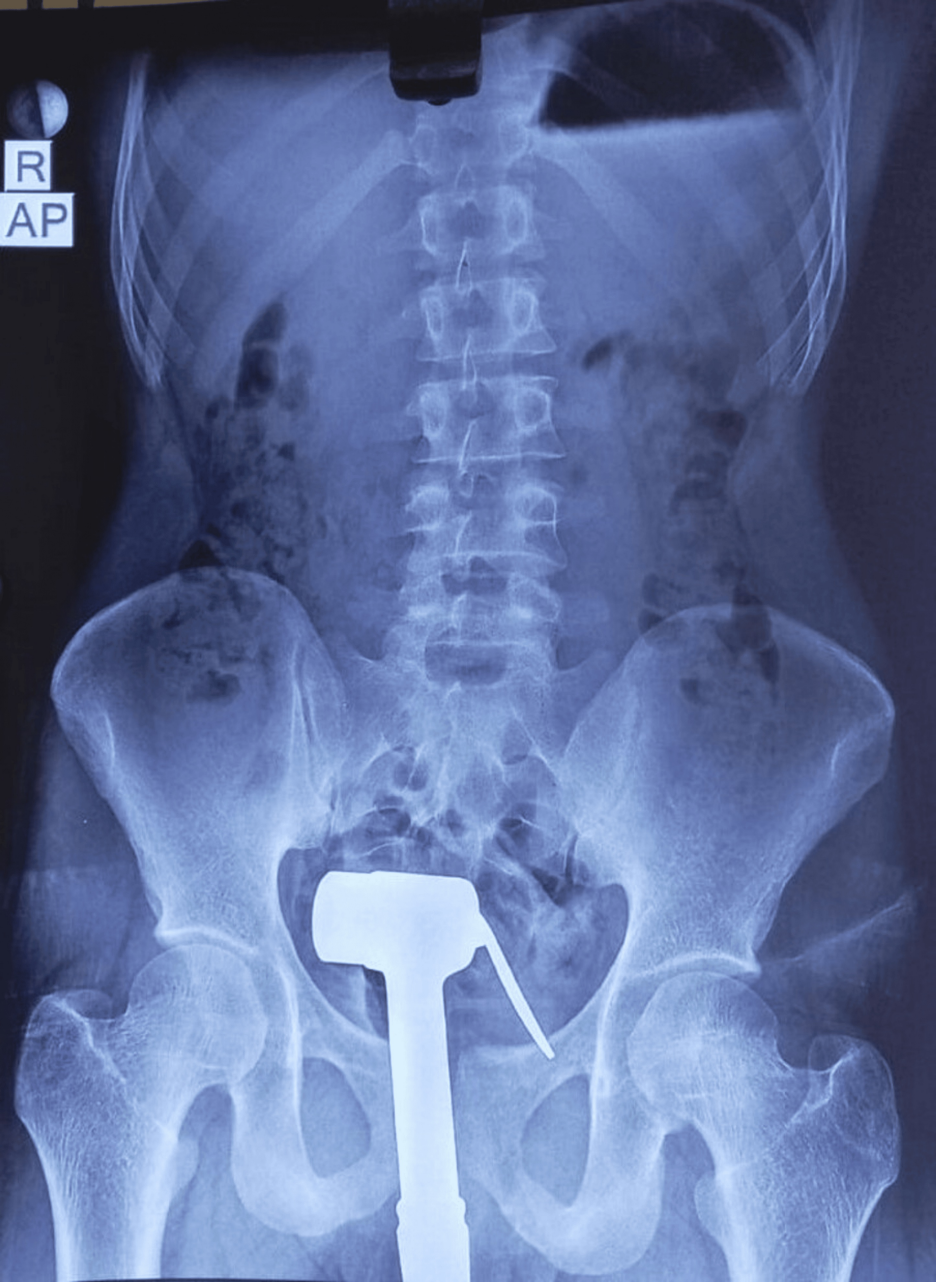
X-ray of the abdomen and pelvis (AP view). AP, anteroposterior

Any attempts at manipulation elicited pain. There was no evidence of blood or any discharge at the orifice and no obvious external injuries. An X-ray abdomen with a pelvis AP view was done to assess the nature, location, and position of the object (Figure 2).

**Figure 3:**
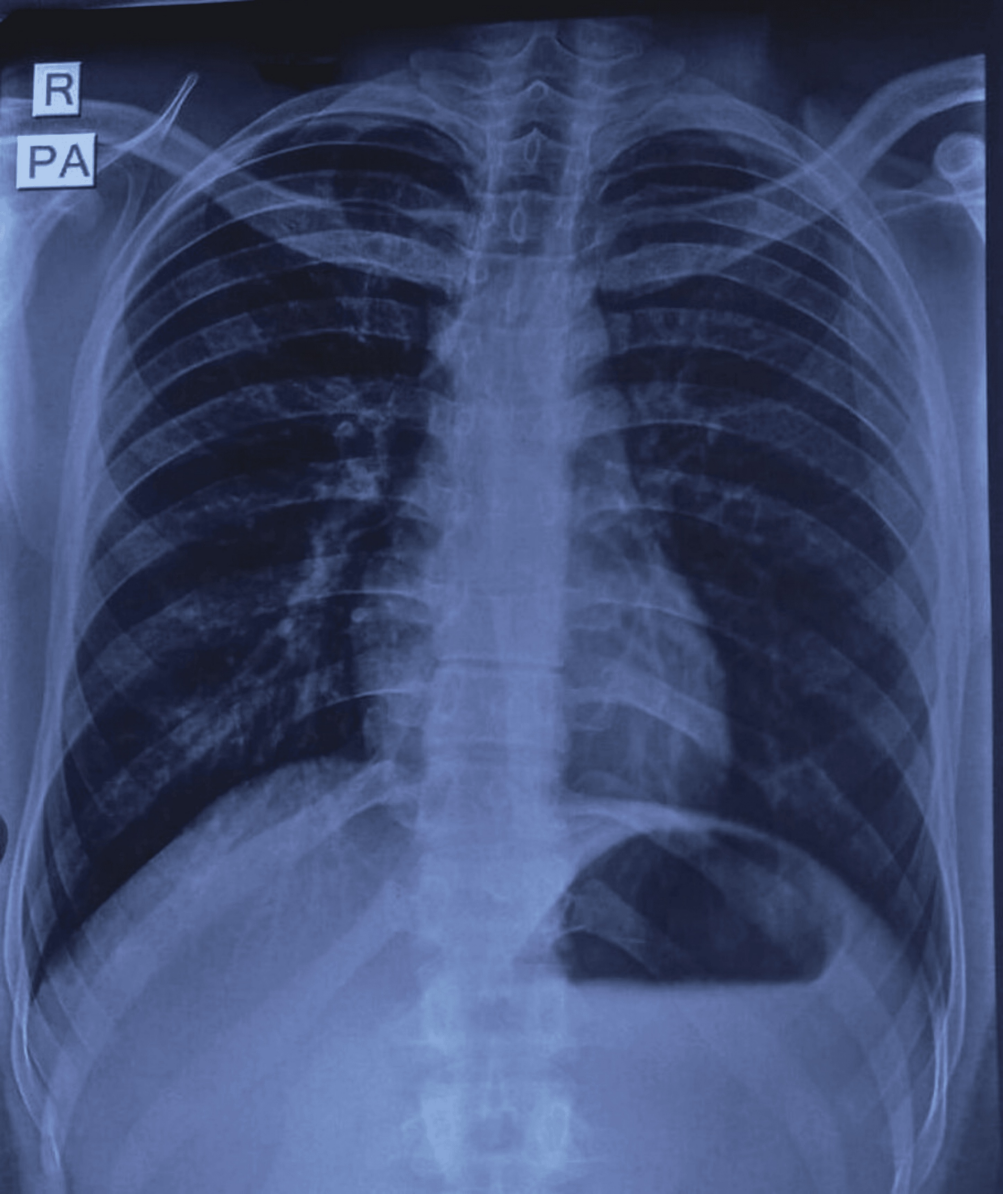
Chest X-ray (PA view). PA, posteroanterior

This revealed the presence of a radiopaque foreign body (shower bidet) within the pelvic cavity, with all its parts intact. The surrounding pelvic bones and vertebrae appeared normal. Chest X-ray (posteroanterior [PA] view) showed no evidence of pneumoperitoneum from a potential perforation caused by the foreign body insertion (Figure 3).

**Figure 4:**
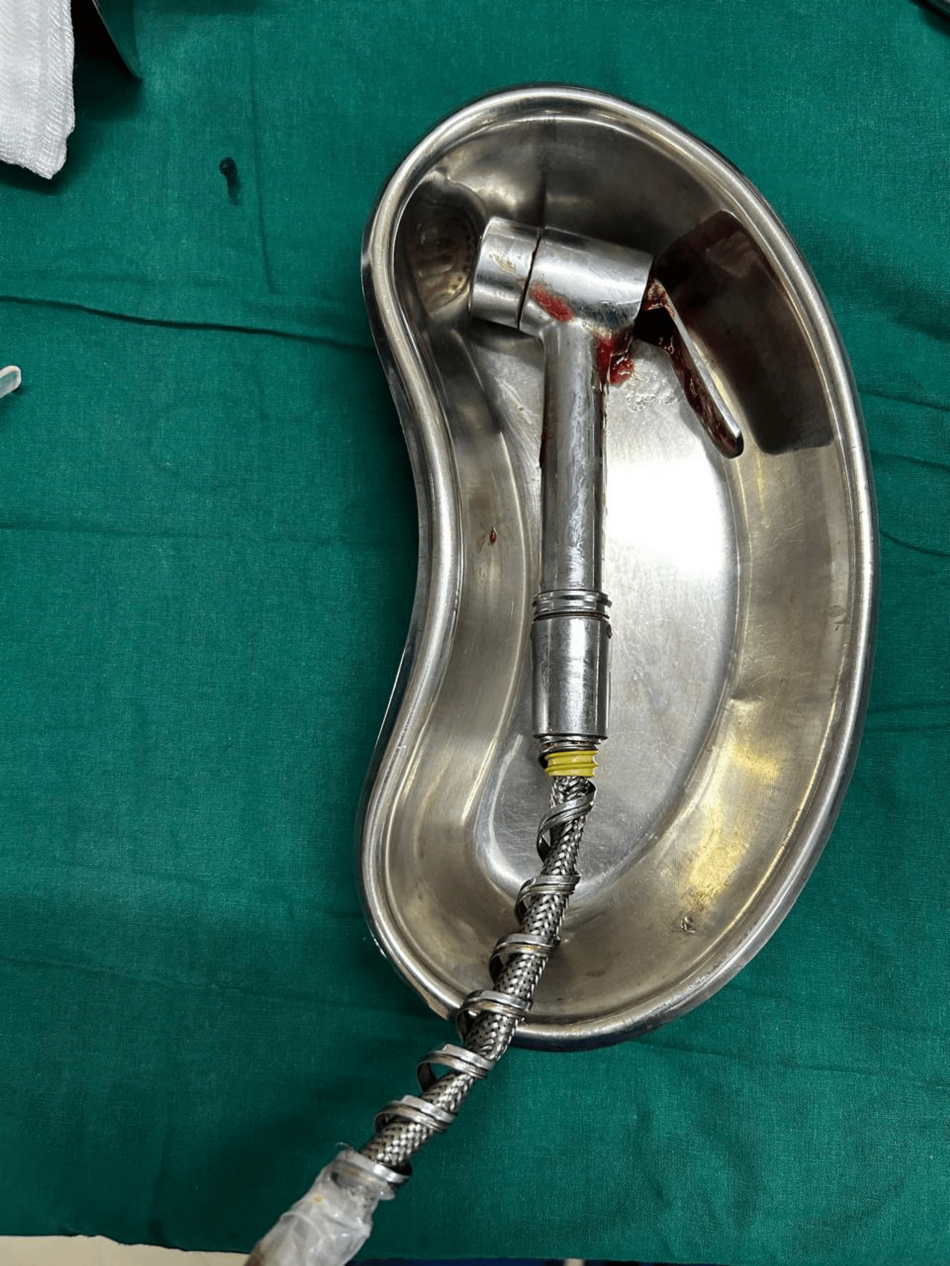
Intact shower bidet retrieved via transanal approach.

After the primary assessment, the decision was made to examine the patient under general anesthesia and perform an exploratory laparotomy if required. He demonstrated comprehension and understanding of his condition and the events that led to it and seemed to appreciate the consequences of this act and potential management strategies. However, he would not repeat and rationalize what was explained to him. Hence, informed consent was given by the father. After administering general anesthesia and achieving muscle relaxation, 2% lignocaine jelly was applied per rectum for the digital rectal examination. Gradual digital anal dilation was done. The fingers could be insinuated between the rectal wall and the handle on the shower bidet. Upon pressing the handle with two fingers and the thumb stabilizing the rest of the bidet, the entire bidet could be removed easily through the anus in toto (Figure 4).

The patient did not develop any complications and experienced minimal blood loss. This can be attributed to the small mucosal tears that the patient may have incurred due to manipulation by himself and his parents at home while attempting to remove the shower bidet. A lignocaine gauze was packed rectally. The recovery of the patient was uneventful, and he was shifted to the recovery room. He was kept under observation overnight, during which a multi-slice plain CT scan with rectal contrast was performed. Mild inflammatory wall thickening of the rectum and rectosigmoid junction was seen, with the rectum, sigmoid colon, and descending colon appearing normal. There was no evidence of a leak of the rectal contrast into the peritoneal cavity, and no radiodense foreign body was noted in the rectum. The patient was then referred to a psychiatrist for a neuropsychological evaluation. He was started on the antipsychotic olanzapine after an assessment; however, he was subsequently lost to follow-up.

## Discussion

It was William Burroughs who stated that it was doubtful whether shame could exist in the absence of sexual libido, bearing testament to the underreporting associated with rectal foreign body insertion, primarily for sexual gratification [1]. Cases of rectal foreign body insertion date back to the 16th century [2], while Smiley first reported such a case in 1919 [5]. Barring anecdotal case reports and some case series, there is a dearth of literature stating the annual incidence of these cases in India, which can be attributed to the social stigma and embarrassment surrounding this. According to a retrospective analysis conducted in the United States, the annual incidence of presentations for rectal foreign bodies increased from 1.2 in 2012 to 1.9 per 100,000 persons in 2021 [3]. This rising trend has been corroborated by Bhasin and Williams showing a bimodal age distribution in the second and fifth decades [1]. Males show a higher incidence than females, with a systematic review documenting a ratio of 37:1 [6]. Anal eroticism in the context of a psychiatric illness, as in our case, further complicates these cases, increasing the chances of recurrence. In a retrospective analysis by O'Farrell et al., 37.5% of patients of rectal foreign bodies had a psychiatric illness [4].

Although psychiatric etiology has been proposed as the underlying mechanism, the prevalence of foreign body insertion/ingestion within this patient population remains undetermined. The insertion of foreign bodies is frequently characterized by repetitive patterns of behavior, potentially manifesting as a response to command hallucinations, particularly in cases of schizophrenia [7].

There are case reports of rectal foreign body insertion in patients with Munchausen’s syndrome, schizophrenia, depressive disorders, Smith-Magenis syndrome, psychosis, and others [8-10]. A case series studying the ingestion and insertion of foreign objects alluded to the psychodynamic formulations leading to this behavior, the details of which are beyond the ambit of this paper. The clinical presentations described in these cases enable deeper analysis through established psychological frameworks. Developmentally, the chosen site of bodily insertion may represent an area of particular psychological significance, potentially indicating arrested development at specific psychosexual stages [11]. The stimulation of the anal canal has been hypothesized as providing temporary relief from psychiatric symptoms by increasing the vagal tone - a well-defined modulator of the brain-gut axis in psychiatric disorders [4,12]. A study utilizing functional MRI for nonpainful anal stimulation revealed activation of different areas of the cerebral cortex [13].

Presentation of cases of rectal foreign body insertions may vary owing to a myriad of factors comprising the motivation leading to this act, voluntary or involuntary, type of object, amount of force involved, and potential delays in presentations to name a few. Patients typically present with abdominal or pelvic pain, constipation, incontinence, bleeding per rectum, and, in rare cases like the present one, an object protruding through the anal orifice. About 20% of patients conceal the history of foreign body insertion [6]. Long-standing cases and those involving sharp objects can also present with sepsis, peritonitis, and hypovolemic shock. When dealing with such patients, an unprejudiced, neutral stance must be taken, putting an embarrassed patient at ease and further building physician-patient trust. The associated social stigma and shame cause delays in patients seeking treatment, with one case report mentioning a hiatus of up to five years [14].

In the context of the aforementioned history and presentation, a strong degree of suspicion must be maintained. The first step would be to rule out acute abdomen and emergency. Hemodynamic instability and abdominal examination with guarding and tenderness may suggest the same. A plain chest X-ray will help detect pneumoperitoneum in case of perforation. An X-ray abdomen with the pelvis aids in assessing the nature, location, and position of the object. This is also especially important to rule out any sharp objects that might pose a risk to the surgeon who might do a blind perrectal examination [15]. A digital rectal examination following this will yield useful information about the proximity of the object concerning the anal verge and pelvic floor. Anal tone and sphincter integrity can also be gauged at this point. Though otherwise unnecessary, laboratory investigations including complete blood count, serum creatinine, and inflammatory markers may be ordered in cases of suspected bowel perforation [16].

Contrast-enhanced CT scan of the abdomen and pelvis will help detect non-radiopaque and organic objects as well as complications such as bowel perforation, abscess formation, or intestinal obstruction. When CT is unavailable, clinicians may obtain either upright or lateral decubitus chest radiographs to check for pneumoperitoneum. While both positions offer similar diagnostic accuracy, patients with peritonitis generally tolerate the lateral decubitus position better. In settings without CT availability, water-soluble contrast enema studies present another diagnostic option, particularly useful for identifying complications like rectal perforation or fistula formation [16]. Small, blunt foreign bodies located in the distal rectum can be attempted to be safely extracted in the Emergency Department. However, objects that are large, sharp, or located higher in the rectum/colon require immediate surgical consultation for removal in the operating room to ensure patient safety [17].

**Figure 5:**
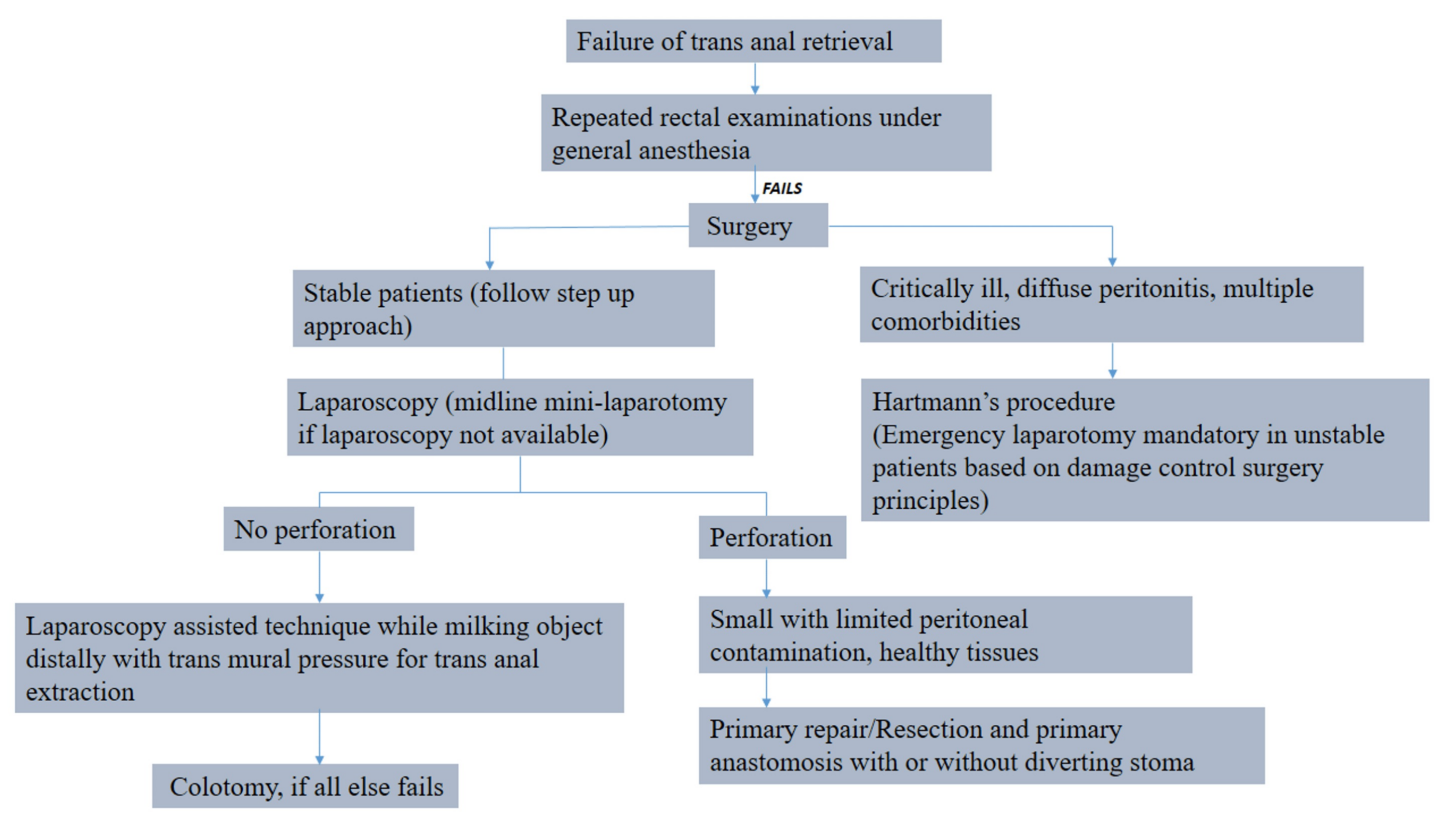
Summary of the WSES-AAST guidelines for a retained rectal foreign body. This is the authors' own creation summarizing the WSES-AAST guidelines for a retained rectal foreign body insertion [17]. WSES-AAST, World Society of Emergency Surgery-American Association for the Surgery of Trauma

Kasotakis et al. described four methods of extraction: the transanal route, endoscopic approach, transabdominal exploration, and, if everything fails, symphysiotomy and removal [18]. The present case was amenable to treatment using the transanal approach under general anesthesia; however, pudendal nerve block and spinal anesthesia are also viable options. This helps put the patient at ease, improves visualization, decreases anal sphincter spasm, and enhances the chances of successful retrieval. This approach has shown to be successful in 74% of cases [6]. There is a discrepancy in the literature regarding post-extraction endoscopy, with some endorsing it, while others have reported a high rate of perforations following the procedure [6,19]. According to the World Society of Emergency Surgery-American Association for the Surgery of Trauma (WSES-AAST) guidelines, high-lying or low-lying objects not retrieved by manual transanal extraction can be approached via rigid or flexible sigmoidoscopy [17]. However, some authors suggest surgery as the first-line management for high-lying, hard, or sharp-edged objects [20]. Lake et al. in their study established that the only determinant statistically related to unsuccessful transanal retrieval is the migration of the foreign body into the sigmoid colon, increasing the operative risk by 2.25-fold [19]. The WSES-AAST guidelines following the failure of transanal extraction have been summarized in the flowchart in Figure 5 [17].

The routine use of antibiotics is not advocated in cases without perforation or infection [17]. Observation periods and follow-up appointments should be individualized based on the etiology and general status of the patient. Psychiatric evaluation, as in the present case, should be done as soon as feasible to avoid recurrences.

## Conclusions

Rectal foreign body insertion, driven by numerous etiologies, remains marred by social stigma. Despite the plethora of presentations, a systematic management algorithm should be followed to avoid further complications. After overcoming the emergent phase and safely extracting the foreign body, the management of these patients requires a holistic, multidisciplinary approach guided by the root cause of the incident. The present case also reveals the reluctance still prevalent in our society to seek help for mental health issues and intellectual disabilities. It is worthwhile to conduct a retrospective analysis of such cases from the past few years to determine sociodemographic features, psychological predispositions, and emerging minimally invasive management strategies.
